# Impact of inhibition in striatal decorrelation of cortical neuronal avalanches

**DOI:** 10.1186/1471-2202-14-S1-P165

**Published:** 2013-07-08

**Authors:** Jovana J Belić, Andreas Klaus, Dietmar Plenz, Jeanette Hellgren Kotaleski

**Affiliations:** 1 Computational Biology, Royal Institute of Technology (KTH), Stockholm, 10691, Sweden; 2 Bernstein Center Freiburg, University of Freiburg, Freiburg, 79104, Germany; 3 Department of Neuroscience, Karolinska Institutet (KI), Stockholm, 17177, Sweden; 4 Section of Critical Brain Dynamics, National Institute of Mental Health (NIH), Bethesda, USA

## 

The Basal Ganglia represent subcortical structures that have a crucial role in determining when a given motor program should be selected and called into action [1]. The input region of the Basal Ganglia (the striatum) contains several distinct cell types. 90-95% of them are medium spiny projection neurons (MSNs) that have high threshold for activation and represent the sole source of the output. There is also a small population of fast-spiking interneurons (FSIs) that receive inputs from a wider range of distinct cortical regions compared to projection neurons [2]. Two sources of GABAergic inhibition onto MSNs are the feedforward inhibition via the FSIs and the feedback inhibition from the axon collaterals of the MSNs themselves. Feedforward inhibition is very powerful and may filter cortical information transmitted by striatal projection neurons [3]. In contrast, feedback inhibition between pairs of MSNs acts predominantly at the distal dendrites, but may still significantly control the overall level of activity of the spiny neurons [4].

We simultaneously recorded local field potentials (LFPs) in the cortex and striatum in order to determine how striatum processes cortical neuronal avalanches. Cortical neuronal avalanches represent activity clusters with a cluster size distribution that follows a power law with exponent -1.5 [5]. Analysis of experimental data revealed that activity clusters in striatum also follow power law distributions, but with an exponent significantly lower than what is observed in the cortex [6]. To understand what controls the LFP statistics observed in experiments, we developed an abstract model of the cortico-striatal network. We investigated to what extent the connectivity pattern between cortex and striatum as well as the inhibition within striatum can explain the experimental results [7]. Our model predicts that striatal inhibition plays a prominent role in shaping the observed striatal dynamics and decorrelating the striatal responses to cortical neuronal avalanches. To understand the contribution of feedforward vs feedback inhibition to the dynamics, we extended our abstract model to spiking networks. We used the model to quantify the role of feedback and feedforward inhibition for decorrelating MSNs, and preliminary results suggest that FSIs play a significant role.

## References

[B1] Grillner S Hellgren Kotaleski J Menard A Saitoh K Wikström M Mechanisms for selection of basic motor programs-roles for the striatum and pallidum Trends in Neuroscience 2005 28 364 370 10.1016/j.tins.2005.05.004 15935487

[B2] Ramanathan J Hanley JJ Deniau JM Bolam JP Synaptic convergence of motor and somatosensory cortical afferents onto GABAergic interneurons in the rat striatum Journal of Neuroscience 2002 22 8158 8169 12223570 10.1523/JNEUROSCI.22-18-08158.2002 PMC6758073

[B3] Mallet N Moine K Charpier S Gonon F Feedforward Inhibition of Projection Neurons by Fast-Spiking GABA Interneurons in the Rat Striatum *In Vivo* The Journal of Neuroscience 2005 25 3857 3869 10.1523/JNEUROSCI.5027-04.2005 15829638 PMC6724938

[B4] Wickens J Arbuthnott G Shindou T Simulation of GABA function in the basal ganglia: computational models of GABAergic mechanisms in basal ganglia function Progress in Brain Research 2007 160 313 329 17499122 10.1016/S0079-6123(06)60018-6

[B5] Beggs J Plenz D Neural avalanches in neocortical circuits The Journal of Neuroscience 2003 23 11167 11177 14657176 10.1523/JNEUROSCI.23-35-11167.2003 PMC6741045

[B6] Klaus A Plenz D Striatal processing of cortical neuronal avalanches 2012 New Orleans, LA: Society for Neuroscience

[B7] Belić J Klaus A Plenz D Hellgren Kotaleski J Neuronal avalanches and the cortico-striatal network BMC Neuroscience 2012 13 Suppl1 P122

